# Economic insecurity during the Great Recession and metabolic, inflammatory and liver function biomarkers: analysis of the UK Household Longitudinal Study

**DOI:** 10.1136/jech-2017-209105

**Published:** 2017-08-28

**Authors:** Claire L Niedzwiedz, Srinivasa Vittal Katikireddi, Aaron Reeves, Martin McKee, David Stuckler

**Affiliations:** 1 Department of Sociology, University of Oxford, Oxford, UK; 2 MRC/CSO Social and Public Health Sciences Unit, University of Glasgow, Glasgow, UK; 3 International Inequalities Institute, London School of Economics and Political Science, London, UK; 4 Department of Public Health and Policy, London School of Hygiene & Tropical Medicine, London, UK; 5 Department of Policy Analysis and Public Management, University of Bocconi, Milan, Italy

**Keywords:** Social Epidemiology, Social Inequalities, Socio-Economic

## Abstract

**Background:**

Economic insecurity correlates with adverse health outcomes, but the biological pathways involved are not well understood. We examine how changes in economic insecurity relate to metabolic, inflammatory and liver function biomarkers.

**Methods:**

Blood analyte data were taken from 6520 individuals (aged 25–59 years) participating in Understanding Society. Economic insecurity was measured using an indicator of subjective financial strain and by asking participants whether they had missed any bill, council tax, rent or mortgage payments in the past year. We investigated longitudinal changes in economic insecurity (remained secure, increase in economic insecurity, decrease in economic insecurity, remained insecure) and the accumulation of economic insecurity. Linear regression models were calculated for nine (logged) biomarker outcomes related to metabolic, inflammatory, liver and kidney function (as falsification tests), adjusting for potential confounders.

**Results:**

Compared with those who remained economically stable, people who experienced consistent economic insecurity (using both measures) had worsened levels of high-density lipoprotein (HDL)-cholesterol, triglycerides, C reactive protein (CRP), fibrinogen and glycated haemoglobin. Increased economic insecurity was associated with adverse levels of HDL-cholesterol (0.955, 95% CI 0.929 to 0.982), triglycerides (1.077, 95% CI 1.018 to 1.139) and CRP (1.114, 95% CI 1.012 to 1.227), using the measure of financial strain. Results for the other measure were generally consistent, apart from the higher levels of gamma-glutamyl transferase observed among those experiencing persistent insecurity (1.200, 95% CI 1.110 to 1.297).

**Conclusion:**

Economic insecurity is associated with adverse metabolic and inflammatory biomarkers (particularly HDL-cholesterol, triglycerides and CRP), heightening risk for a range of health conditions.

## Background

Perceived economic insecurity is linked to poor health, including depressive and anxiety disorders, diabetes and coronary heart disease,^1–4^ as well as hazardous health behaviours. It appears to play an important mediating role in the relationship between adversity (such as job loss or social disadvantage) and health and well-being.^5–7^ Indeed, fear of job loss can be just as harmful as, if not more than, the job loss itself.^8–10^ Although a positive correlation between economic insecurity and overall ill health is well-established, the biological pathways through which these operate are not well understood.

Economic insecurity may impact on metabolic, inflammatory and liver functions. This may result from changes in health-related behaviours as well as psychosocial stress. Perceived economic insecurity may trigger increased alcohol consumption, as a coping mechanism, which may manifest biologically through changes in gamma-glutamyl transferase (GGT), a liver enzyme that metabolises drugs and other toxins. GGT has been implicated, along with oxidative stress and inflammation pathways, in diseases such as type II diabetes and cardiovascular disease.^11 12^ In contrast, economic insecurity may result in decreased alcohol consumption, as individuals find alcohol unaffordable. While overall levels of alcohol consumption fell during the recent economic crisis, hazardous binge drinking increased among existing heavy drinkers.^13 14^ Similarly, economic insecurity may result in the increased consumption of unhealthy foods, which could impact on metabolic markers, such as cholesterol, triglycerides and glycated haemoglobin (HbA1c). However, although plausible, recent longitudinal evidence indicates unhealthy behaviours do not play a major role in mediating the relationship between perceived financial strain and self-reported health, suggesting economic insecurity could affect health via other mechanisms.^15^

It is also possible that changes in economic insecurity operate directly via psychosocial pathways, for example, stress may act on inflammatory responses, metabolic pathways and regulation of cardiovascular systems.^15–18^ Psychosocial stress affects the hypothalamic–pituitary–adrenocortical axis and the sympathetic–adrenal–medullary system, which regulate a range of processes including inflammatory responses, gluconeogenesis and the metabolism of carbohydrates and fats.^17 19^ It has also been suggested that inflammatory markers, such as fibrinogen, may only be cardiotoxic when combined with certain psychosocial stressors including financial strain, but not depressive symptoms.^20^ Additionally, economic insecurity may result in poor mental health, which could act on the inflammatory system, but recent longitudinal research has questioned this causal pathway suggesting that inflammation (as measured by C reactive protein (CRP)) may result in psychological distress, or that there may be bidirectional relationships.^16 21^ Previous work has found that the threat of redundancy or job insecurity increases certain biomarkers, such as cholesterol,^9 22^ and that an adverse psychosocial working environment, including the experience of organisational changes, is associated with increased HbA1c, a marker of blood glucose levels.^23^

If economic insecurity has a direct impact on biological systems due to psychosocial stress, we might expect it to have the strongest associations with stress-related biomarkers (eg, inflammatory and metabolic markers). Consistent with this, evidence suggests perceived economic insecurity directly impacts oxidative stress (the imbalance of oxidant and antioxidant defences)^24^ and inflammatory markers.^20 25^ Acute stress is also associated with a range of metabolic markers, such as high-density lipoprotein (HDL)-cholesterol and triglycerides, suggestive of a direct link.^26 27^ Therefore, economic insecurity may affect inflammatory and metabolic biomarkers through both psychosocial stress and health-behaviour pathways, with liver function primarily hypothesised to operate via alcohol consumption. However, few studies have examined the relationship between economic insecurity and metabolic, inflammatory and liver function biomarkers. Existing studies are often cross-sectional, with potential for reverse causality, and cover one or a limited selection of many possible biomarkers,^24 25 28^ partly owing to a lack of available data. Biomarkers may differ in their sensitivity to economic insecurity. For example, inflammatory markers, which can fluctuate rapidly, may be more responsive in the short term than measures of kidney function, which more likely reflects long-term, cumulative exposure to economic insecurity. In addition, existing studies are often not representative of populations, confined to particular ethnic groups, older adults or occupational cohorts.^9 24 25 29 30^

In this study, we address these limitations using a rich, population-representative source of longitudinal data, the UK Household Longitudinal Study (Understanding Society). We explore how economic insecurity is related to metabolic, inflammatory and liver function biomarkers derived from blood analyte data. Specifically, we test two main hypotheses:

- Do short-term changes in economic security relate to more adverse metabolic, inflammatory and liver function biomarkers?
- Is there a potential cumulative effect of economic insecurity on these biomarkers?

To provide specificity we also investigate biomarkers linked to kidney function, which should not correlate with changes in economic insecurity in the short term (a ‘falsification test’). The biomarkers used in the study are listed in table 1.

**Table 1:** Description of biomarkers included in the study

Type of biomarker	Specific biomarker (method of measurement, units/lowest detection limit)	Function and clinical significance
*Inflammatory*	High-sensitivity C reactive protein (CRP) (analysed from serum using the N Latex CRP mono immunoassay on the Behring Nephelometer II analyser, with lowest detection limit of 0.2 mg/L)	CRP is an acute-phase reactant produced by the liver and is indicative of general inflammation, which may be present due to infection or chronic disease. Elevated levels are also related to psychological distress.
	Fibrinogen (analysed from citrate plasma samples using a modification of the Clauss thrombin clotting method on the IL-ACS-TOPS analyser, with lowest detection limit of 0.5 g/L)	Fibrinogen is also produced by the liver and is involved in the blood clotting cascade. Higher levels are associated with heightened risk of cardiovascular disease.
*Metabolic function*	Total cholesterol (measured from blood serum using enzymatic methods with a Roche Modular P analyser calibrated to the Centers for Disease Control and Prevention guidelines, mmol/L)	Total cholesterol is the overall level of cholesterol in the blood, which is transported through the bloodstream by lipoproteins: low-density lipoprotein (LDL)-cholesterol and high-density lipoprotein (HDL)-cholesterol (see below). LDL-cholesterol contributes to plaques that are deposited in the arteries, rendering them less flexible and leading to atherosclerosis and an increased risk of myocardial infarction and stroke. In addition to HDL and LDL cholesterol, triglycerides (see below) contribute to the total blood cholesterol level.
	HDL-cholesterol (same as total cholesterol above, mmol/L)	HDL-cholesterol is involved in the delivery of LDL-cholesterol from the arteries to the liver, where it is broken down. Higher levels are therefore beneficial for the body and protective against cardiovascular disease.
	Triglycerides (blood samples were collected from participants who did not fast and measured from serum blood using an enzymatic method, on a Roche P module analyser, mmol/L)	Triglycerides are lipids that are used to store excess energy from the diet. Higher levels may arise as a result of obesity and other lifestyle-related risk factors, which increase the risk of cardiovascular disease.
	Glycated haemoglobin (HbA1c) (measured from whole blood using high-performance liquid chromatography cation exchange on a Tosoh G8 analyser, mmol/mol)	HbA1c measures glucose intolerance. HbA1c forms when high circulating levels of glucose attach themselves to the haemoglobin molecule. HbA1c levels are reflective of control of blood glucose over the previous 8–12 weeks and can be used to assist in the diagnosis of diabetes. An HbA1c level of 48 mmol/mol (6.5%) is recommended as the cut-off point for diabetes.
*Liver function*	Gamma-glutamyl transferase (GGT) (measured with an enzymatic method on the Roche P module analyser, with lowest detection limit of 5 μ/L)	GGT is an enzyme contained in liver cells (and others such as kidney, bile duct and pancreas) and is involved in the metabolism of drugs and toxins. It is the most sensitive measure of alcohol consumption and liver damage, and elevated levels are related to cardiovascular disease.
*Kidney function*	Urea (measured from serum samples with a kinetic ultraviolet assay on a Roche P module analyser, mmol/L)	Urea is a waste product formed from the breakdown of proteins, which is excreted via urine. Elevated levels indicate that the kidneys are not functioning properly, which may be due to acute or chronic kidney disease.
	Creatinine (measured from serum samples using an enzymatic method on the Roche P module analyser, μmol/L)	Creatinine is a chemical waste product of muscle breakdown, which is excreted by the kidneys and flows into the urine. Creatinine is therefore an indicator of how well the kidneys are cleaning the blood and is usually a more accurate measure of kidney function compared with urea.

## Methods

### Data

We analysed data from the UK Household Longitudinal Study.^31^ It is a survey of approximately 40 000 households and includes a General Population Survey (GPS) sample, as well the former British Household Panel Survey (BHPS) sample. Data were collected through face-to-face interviews and a self-completion questionnaire from adults aged 16 years and over within recruited households. Health assessments were conducted by nurses for the GPS (wave 2, 2010/2011) and former BHPS (wave 3, 2011/2012) samples.^32^ These data were integrated with relevant longitudinal mainstage data.

Of the wave 2 GPS sample, 72.94% (n=26 961) were eligible for the nurse visit, 15 591 responded and 10 175 individuals agreed to provide a blood sample. Among the BHPS sample, 78.43% (n=8914) individuals were eligible for a nurse visit, 5053 responded and 3342 individuals agreed to a blood sample.

Combining the GPS and BHPS blood samples provided a total of 13 517 individuals, and of these 13 107 samples were successfully processed for at least one biomarker. Our analysis focuses on individuals (n=7464) who were of core working-age (25–59 years). Further information about the data collection process and sample has been published elsewhere.^33 34^

### Biomarkers

We investigated the following biomarkers derived from the blood analyte samples: inflammatory markers (CRP and fibrinogen), metabolic markers (total cholesterol, HDL-cholesterol, triglycerides and HbA1c) and a measure of liver function which can be used to indicate alcohol consumption (GGT). As falsification tests we also selected urea and creatinine, measures of kidney function, which we did not expect to be causally related to a short-term change in economic security, to examine the specificity of the associations. Further details of the biomarker data collection is found in online supplementary additional file 1 and elsewhere.^33^

10.1136/jech-2017-209105.supp1 Supplementary data

### Exposures

Economic security was measured by two indicators of subjective financial circumstances. Participants were asked how well they felt they were managing financially at both points (hereafter referred to as financial strain): living comfortably (0), doing alright (0), just about getting by (1), finding it quite difficult (2) or finding it very difficult (2). The living comfortably and doing alright categories were combined to form an economically secure group, as were the just about getting by, finding it quite and very difficult groups, forming an economically insecure group. Participants were then categorised into the following groups: remained economically secure; experienced an increase in economic insecurity; experienced a decrease in economic insecurity; and remained economically insecure. A second measure of economic security was derived from questionnaire items asking participants about whether they were up to date with their bills or behind with council tax, rent or mortgage payments in the past year (hereafter referred to as missed bills). Respondents were then grouped into the same categories as the other measure of economic security above. In addition to these change variables, we also calculated a measure of cumulative economic insecurity, which ranged from 0 to 4. The scores for the financial strain measure are shown in brackets above. For the missed bills measure, we added up the number of times participants reported falling behind with the three types of bills at both time points, top-coding at four due to the small number of people who experienced problems five or more times.

### Covariates

We considered the following potential sociodemographic confounding variables, measured at baseline, on the basis of prior understanding: age (years), sex, education level categorised into low (no qualifications, General Certificate of Secondary Education), medium (A level) or high (degree/other higher degree), marital status (married/cohabiting, single, divorced/separated/widowed), employment status (currently in paid employment vs not), social class (using the National Statistics Socio-economic Classification (NS-SEC)), and equivalised gross monthly household income divided into quintiles. We use the five-class version of the NS-SEC, but also include those who had never worked or were long-term unemployed in an ‘other’ category. Individuals with missing social class data were imputed from their partner where possible (n=100). Long-standing illness or impairment (yes vs no), self-reported health (good vs poor) and psychological distress were also considered as baseline health-related confounding factors. Psychological distress was measured using General Health Questionnaire (GHQ) caseness (using a score of 4 or more as the cut-off).^35^ These potential confounding factors may influence economic insecurity and are likely to be associated with the biomarkers under study. As potential mediating variables, we included body mass index (BMI) (below 25/25 to 30/above 30), derived from height and weight measurements taken during the nurse visit at the second time point. BMI was only available for the GPS sample at baseline and was based on self-reported measurements. Ideally, we could have included further health-related behaviour data, but these had limited availability. However, we were able to include current smoking status (measured as whether the respondent reported smoking in the previous 24 hours), which was also collected during the nurse visit.

### Statistical analysis

We first calculated descriptive statistics for each outcome variable and the exposure variables. All outcome variables were logged, as most had a positive skew and, otherwise, for consistency. Therefore, the geometric means for the outcome variables are presented. Linear regression models for each biomarker outcome were calculated with the change in economic insecurity variable, age and sex included as covariates as follows:

$$log(biomarker_{i})=\beta_{0}+\beta_{1}insecurity_{i}+\beta_{2}age_{i}+\beta_{3}sex_{i}+\epsilon_{i}$$

Here *i* is the individual, insecurity refers to a set of dummy variables for the individual’s change in economic security between the two time points under study, age is the individual’s age in years at baseline, and sex is a dummy variable. The exponentiated regression coefficients were calculated, and these correspond to the change in the ratio of the expected geometric mean of the original outcome variable.^36^ For example, if the linear regression coefficient for the dummy variable ‘sex’ above was equal to 0.12 for women, the exponentiated coefficient would be equal to 1.13. Compared with men, we would therefore expect women to have 13% higher geometric mean values for the biomarker under study, when the other variables are held constant.^37^

We added covariates in stages: first, sociodemographic confounding variables (education level, marital status, employment status and social class); second, household income; third, limiting long-term illness and self-reported health; and fourth, GHQ caseness. We repeated the above analyses using the measure of economic insecurity derived from self-reported missed bill payments. As the results were generally consistent using the two measures, we primarily report results for the measure of financial strain, with results for the other measure available in online supplementary additional file 2. Additionally, we calculated fully adjusted models mutually adjusting for the two measures of economic insecurity to see if independent associations were apparent and using the cumulative measure of economic insecurity.

The descriptive statistics and statistical models were weighted to account for selection into the blood sample group, and take into account the complex structure of the survey. Respondents with missing data on the exposure variables were excluded from the analysis (n=944, 12.65%). Individuals with complete data for at least one biomarker were included in the sample; most people had complete data for all outcome variables (n=6026, 80.73%; online supplementary additional file 2). Each model calculated, therefore, contains a different number of individuals depending on the number of missing cases. As recommended,^33^ individuals with CRP values >10 mg/L (n=290) were excluded in the analysis of this parameter, as elevated values are indicative of bacterial and viral infections, rather than chronic inflammation which would plausibly be related to psychosocial stress.^38^ Several sensitivity analyses were performed (see below). All analyses were performed using Stata SE V.14.1.

## Results

The analysis included 6520 individuals (56.1% female) (table 2, with further descriptive statistics reported in online supplementary additional file 2). Individuals who reported persistent economic insecurity generally had the most adverse biomarker outcomes, in models adjusting for age and sex (table 3, model 1). Specifically, persistent economic insecurity was most strongly associated with elevated CRP. Compared with those who remained secure, CRP levels were 30.8% higher (1.308, 95% CI 1.226 to 1.395). Among those reporting an increase in economic insecurity, CRP levels were elevated, but not to the same extent as the consistently insecure group, and levels were not elevated among the group experiencing a decrease in insecurity. HDL-cholesterol, triglyceride, GGT, fibrinogen and HbA1c levels were also most adverse among the persistently insecure group. Total cholesterol did not differ across these groups. Creatinine and urea levels were also lower among the group who experienced persistent insecurity.

**Table 2:** Descriptive statistics for the sample (weighted percentages)

Variable	N	%
Age (mean, SD)	42.19	9.74
Sex		
Male	2742	43.9
Female	3778	56.1
Economic security (financial strain)		
Remained secure	3264	46.6
Increased insecurity	715	11.1
Decreased insecurity	644	10.2
Remained insecure	1897	32.0
Economic security (missed bills)		
Remained secure	5093	75.1
Increased insecurity	444	7.0
Decreased insecurity	507	8.7
Remained insecure	476	9.2
Highest educational qualification		
Low	2509	39.0
Medium	1318	20.2
High	2693	40.8
Marital status		
Single	746	15.0
Married/cohabiting	4943	74.2
Divorced/separated/widowed	831	10.9
Employment status		
Not employed	1292	21.6
Employed	5228	78.4
Social class (NS-SEC)		
Management, administrative and professional	2391	34.3
Intermediate	759	11.1
Small employers and own account workers	479	7.0
Lower supervisory and technical	407	6.3
Semi-routine and routine	1106	18.4
Other	1378	23.0
Household income quintile		
1	1591	26.6
2	1631	25.9
3	1652	24.0
4	1646	23.5
Long-standing illness or impairment		
Yes	2199	32.7
No	4321	67.3
Self-reported health		
Good	5128	78.3
Poor	1392	21.7
GHQ (caseness)		
No	5419	82.0
Yes	1101	18.0
Current smoking		
No	6059	92.1
Yes	461	7.9
Body Mass Index		
Below 25	2023	31.9
25 and below 30	2509	38.1
Above 30	1988	30.0
Total	6520	100.0

**Table 3:** The association between changes in economic insecurity (using financial strain) and metabolic, inflammatory, liver and kidney function biomarkers in Understanding Society

	Total cholesterol	HDL-cholesterol	Triglycerides	HbA1c	CRP	Fibrinogen	GGT	Creatinine	Urea
	Coefficient	Coefficient	Coefficient	Coefficient	Coefficient	Coefficient	Coefficient	Coefficient	Coefficient
	[95% CI]	[95% CI]	[95% CI]	[95% CI]	[95% CI]	[95% CI]	[95% CI]	[95% CI]	[95% CI]
**Model 1**									
Increased insecurity†	0.999	0.942***	1.099**	1.010	1.180***	1.020	1.061	1.000	0.996
	[0.980 to 1.019]	[0.916 to 0.968]	[1.039 to 1.163]	[0.996 to 1.024]	[1.071 to 1.300]	[0.999 to 1.042]	[0.991 to 1.135]	[0.985 to 1.016]	[0.975 to 1.018]
Decreased insecurity	1.004	0.959**	1.074*	1.020*	1.094	1.025*	1.050	0.988	0.987
	[0.984 to 1.024]	[0.933 to 0.987]	[1.016 to 1.136]	[1.004 to 1.036]	[0.992 to 1.207]	[1.004 to 1.047]	[0.985 to 1.119]	[0.971 to 1.005]	[0.964 to 1.010]
Remained insecure	1.001	0.916***	1.111***	1.048***	1.308***	1.053***	1.131***	0.986*	0.959***
	[0.987 to 1.015]	[0.898 to 0.934]	[1.070 to 1.153]	[1.035 to 1.061]	[1.226 to 1.395]	[1.039 to 1.068]	[1.080 to 1.184]	[0.974 to 0.997]	[0.942 to 0.977]
**Model 2**									
Increased insecurity†	1.000	0.947***	1.086**	1.005	1.139**	1.014	1.037	1.004	1.000
	[0.981 to 1.019]	[0.921 to 0.974]	[1.027 to 1.149]	[0.992 to 1.019]	[1.034 to 1.256]	[0.993 to 1.036]	[0.972 to 1.107]	[0.988 to 1.019]	[0.979 to 1.022]
Decreased insecurity	1.004	0.967*	1.057	1.013	1.040	1.018	1.019	0.993	0.993
	[0.985 to 1.024]	[0.940 to 0.995]	[0.999 to 1.118]	[0.997 to 1.030]	[0.941 to 1.150]	[0.996 to 1.039]	[0.954 to 1.088]	[0.976 to 1.010]	[0.970 to 1.018]
Remained insecure	1.004	0.928***	1.079***	1.037***	1.208***	1.039***	1.071**	0.994	0.974**
	[0.990 to 1.018]	[0.910 to 0.947]	[1.038 to 1.122]	[1.024 to 1.049]	[1.126 to 1.296]	[1.024 to 1.054]	[1.021 to 1.124]	[0.982 to 1.006]	[0.957 to 0.991]
**Model 3**									
Increased insecurity†	1.000	0.950***	1.088**	1.005	1.130*	1.010	1.034	1.007	1.004
	[0.981 to 1.020]	[0.924 to 0.977]	[1.028 to 1.151]	[0.992 to 1.019]	[1.024 to 1.247]	[0.989 to 1.032]	[0.968 to 1.104]	[0.992 to 1.022]	[0.983 to 1.026]
Decreased insecurity	1.005	0.971*	1.059*	1.013	1.030	1.013	1.015	0.997	0.998
	[0.985 to 1.026]	[0.944 to 1.000]	[1.001 to 1.120]	[0.997 to 1.030]	[0.931 to 1.139]	[0.991 to 1.035]	[0.950 to 1.085]	[0.980 to 1.014]	[0.975 to 1.023]
Remained insecure	1.005	0.934***	1.083***	1.037***	1.194***	1.032***	1.065*	1.000	0.981*
	[0.990 to 1.020]	[0.914 to 0.954]	[1.039 to 1.128]	[1.024 to 1.050]	[1.109 to 1.285]	[1.017 to 1.048]	[1.014 to 1.118]	[0.988 to 1.013]	[0.963 to 0.999]
**Model 4**									
Increased insecurity†	1.001	0.955**	1.078**	1.001	1.114*	1.008	1.017	1.007	1.005
	[0.982 to 1.021]	[0.929 to 0.982]	[1.019 to 1.140]	[0.987 to 1.014]	[1.011 to 1.226]	[0.987 to 1.029]	[0.954 to 1.085]	[0.992 to 1.023]	[0.983 to 1.027]
Decreased insecurity	1.006	0.975	1.051	1.009	1.015	1.010	1.003	0.997	0.999
	[0.986 to 1.027]	[0.948 to 1.003]	[0.994 to 1.112]	[0.993 to 1.025]	[0.919 to 1.120]	[0.989 to 1.032]	[0.940 to 1.071]	[0.980 to 1.014]	[0.975 to 1.023]
Remained insecure	1.007	0.944***	1.060^**^	1.026***	1.147***	1.025**	1.028	1.001	0.983
	[0.992 to 1.023]	[0.924 to 0.964]	[1.017 to 1.105]	[1.014 to 1.038]	[1.065 to 1.236]	[1.010 to 1.041]	[0.978 to 1.080]	[0.988 to 1.013]	[0.965 to 1.001]
**Model 5**									
Increased insecurity†	1.001	0.955**	1.077**	1.001	1.114*	1.008	1.017	1.007	1.005
	[0.982 to 1.021]	[0.929 to 0.982]	[1.018 to 1.139]	[0.987 to 1.014]	[1.012 to 1.227]	[0.987 to 1.029]	[0.953 to 1.084]	[0.992 to 1.023]	[0.983 to 1.027]
Decreased insecurity	1.005	0.976	1.045	1.009	1.017	1.010	1.000	0.997	1.000
	[0.985 to 1.026]	[0.948 to 1.004]	[0.988 to 1.106]	[0.993 to 1.026]	[0.922 to 1.123]	[0.989 to 1.032]	[0.937 to 1.067]	[0.980 to 1.014]	[0.977 to 1.025]
Remained insecure	1.006	0.945***	1.052*	1.026***	1.152***	1.026**	1.023	1.000	0.984
	[0.991 to 1.022]	[0.925 to 0.965]	[1.009 to 1.097]	[1.014 to 1.039]	[1.068 to 1.242]	[1.010 to 1.042]	[0.973 to 1.075]	[0.987 to 1.013]	[0.966 to 1.003]
N	6425	6417	6427	6162	6003	5905	6390	6437	6439

Linear regression models calculated using logged dependent variables, with exponentiated coefficients shown. 95% CIs in brackets.

Model 1: adjusted for age and sex; model 2: model 1 + education level, marital status, employment status, social class; model 3: model 2 + household income quintile; model 4: model 3 + limiting long-standing illness, self-reported health; model 5: model 4 + GHQ caseness.

*p<0.05, **p<0.01, ***p<0.001.

†Reference is economically secure at both time points.

CRP, C reactive protein; GGT, gamma-glutamyl transferase; GHQ, General Health Questionnaire; HbA1c, glycated haemoglobin.

**Figure 1:**
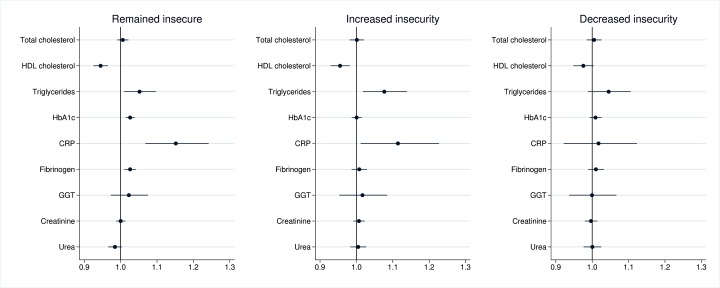
Economic insecurity (using financial strain) and metabolic, inflammatory, liver and kidney function biomarkers (coefficients are exponentiated). CRP, C reactive protein; GGT, gamma-glutamyl transferase; HbA1c, glycated haemoglobin; HDL, high-density lipoprotein.

Adjustment for the first set of potential confounding variables (baseline education level, marital status, employment status and social class) attenuated a number of the relationships (table 3, model 2). The observed associations in the persistently insecure group remained, but were weakened; CRP levels were 20.8% higher than those who felt secure at both time points. Reporting increased economic insecurity continued to be associated with adverse patterns of HDL-cholesterol, triglycerides and CRP. Reporting decreased insecurity was only related to adverse HDL-cholesterol levels, but not to the same extent as the groups that remained insecure or felt increased insecurity. Adjusting for household income modestly attenuated most of the associations. Further adjustment for baseline health status (table 3, model 4) resulted in further attenuation. Most notably, GGT was no longer elevated among those who felt insecure at both time points. Adjustment for GHQ also attenuated the coefficients (table 3, model 5, and figure 1). However, the group that remained insecure still exhibited adverse levels of HDL-cholesterol, triglycerides, CRP, HbA1c and fibrinogen, compared with those experiencing no insecurity. In addition, increased insecurity was also related to more adverse levels of HDL-cholesterol, triglycerides and CRP.

The results were generally consistent for the measure of economic insecurity derived from reports of missed bill payments (see online supplementary additional file 2, table A5). The main difference that was apparent concerned GGT; higher levels were found among the consistently insecure group, which persisted when adjusting for all the potential confounding factors. In models that adjusted both measures of economic insecurity, GGT remained significantly associated with persistent missed bill payments (see online supplementary additional file 2).

Moving to the cumulative measure of economic insecurity, we found increased HbA1c, CRP and fibrinogen levels as the extent of economic insecurity increased according to the cumulative measure of financial strain (table 4). HbA1c levels were 4.9% (1.049, 95 % CI 1.017 to 1.082) higher in those scoring 4 on the measure, compared with those with no experience of economic insecurity. This was less apparent for triglycerides and HDL-cholesterol. Results were mixed for the other measure of economic insecurity, with the most apparent evidence of a potential cumulative effect observed for GGT (see online supplementary additional file 2).

**Table 4:** The association between the cumulative measure of economic insecurity (using financial strain) and metabolic, inflammatory, liver and kidney function biomarkers in Understanding Society

	Total cholesterol	HDL-cholesterol	Triglycerides	HbA1c	CRP	Fibrinogen	GGT	Creatinine	Urea
	Coefficient	Coefficient	Coefficient	Coefficient	Coefficient	Coefficient	Coefficient	Coefficient	Coefficient
	[95% CI]	[95% CI]	[95% CI]	[95% CI]	[95% CI]	[95% CI]	[95% CI]	[95% CI]	[95% CI]
1†	1.001	0.964**	1.072**	1.002	1.077	1.008	0.994	1.003	1.001
	[0.985 to 1.017]	[0.942 to 0.986]	[1.025 to 1.122]	[0.990 to 1.014]	[0.994 to 1.168]	[0.990 to 1.026]	[0.943 to 1.047]	[0.990 to 1.016]	[0.982 to 1.019]
2	1.003	0.960***	1.018	1.017**	1.080	1.021*	1.026	1.006	0.993
	[0.987 to 1.020]	[0.938 to 0.983]	[0.970 to 1.067]	[1.004 to 1.030]	[0.996 to 1.172]	[1.004 to 1.038]	[0.970 to 1.085]	[0.992 to 1.020]	[0.971 to 1.015]
3	1.022	0.923***	1.111**	1.031**	1.208***	1.029*	1.066	0.995	0.983
	[0.999 to 1.047]	[0.894 to 0.953]	[1.043 to 1.182]	[1.012 to 1.050]	[1.080 to 1.352]	[1.004 to 1.054]	[0.989 to 1.148]	[0.976 to 1.013]	[0.956 to 1.010]
4	0.992	0.943**	1.046	1.049**	1.240***	1.034*	0.993	0.987	0.980
	[0.964 to 1.022]	[0.904 to 0.984]	[0.975 to 1.123]	[1.017 to 1.082]	[1.095 to 1.406]	[1.006 to 1.063]	[0.908 to 1.085]	[0.960 to 1.014]	[0.945 to 1.016]
N	6385	6377	6387	6124	5963	5866	6350	6396	6398

Linear regression models calculated using logged dependent variables, with exponentiated coefficients shown. 95% CI in brackets.

Model adjusted for age, sex, education level, marital status, employment status, social class, household income quintile, limiting long-standing illness, self-reported health and GHQ caseness.

*p<0.05, **p<0.01, ***p<0.001.

†Reference is no economic insecurity.

CRP, C reactive protein; GGT, gamma-glutamyl transferase; GHQ, General Health Questionnaire; HbA1c, glycated haemoglobin; HDL, high-density lipoprotein.

### Sensitivity analyses

We restricted the analysis excluding individuals who reported taking statins, anti-inflammatory medication, contraception or hormone replacement therapy in the past 7 days prior to the nurse visit (n=828). We also calculated additional models adjusted for smoking status and BMI to see if the associations persisted. We repeated our statistical models restricting the sample to those with complete data for all biomarker outcomes (n=5438). None of these additional analyses substantively changed our results (see online supplementary additional file 2).

## Discussion

### Summary of findings

Our study reveals that economic security is associated with several different biomarkers linked to greater health risk. Biomarkers related to adverse metabolic and inflammatory situations, including HDL-cholesterol, triglycerides and CRP, appear particularly sensitive. An important finding was the relative consistency in the pattern of results between both measures of economic security and the persistence of several associations when adjusting for a range of potential sociodemographic and health-related confounding factors.

Our results provide some clues to understanding potential biological pathways arising from economic insecurity. The clearest associations were seen for CRP, which is more closely linked to the inflammatory pathway than fibrinogen (which also has a key role in the blood clotting cascade). This provides some support for the role of inflammation in embodying the adverse health impacts of economic insecurity, possibly through a psychosocial stress pathway. Results for triglycerides and HDL-cholesterol suggest that health-related behaviours, particularly diet, could also be important in addition to psychosocial stress, but this merits further exploration. Higher HDL-cholesterol levels are thought to be protective of cardiovascular disease. Thus, lower levels of HDL-cholesterol and higher triglyceride levels could be implicated in a biological pathway linking economic insecurity to cardiovascular disease.^39 40^ Alcohol consumption, as reflected by GGT, was associated with the measure of economic insecurity relating to missed bill payments, persisting after mutual adjustment for the measure of financial strain and also BMI and smoking. Self-reported missed bill payments may reflect a slightly more objective measure of economic insecurity. This particular finding could be due to changing drinking patterns, including increased binge drinking, in response to economic insecurity, but requires further exploration using a more accurate measure of objective economic security that could be compared with subjective measures.

Our results demonstrate that experiencing economic insecurity is related to adverse metabolic and inflammatory biomarker outcomes, but it is much less clear whether present or past insecurity matters more. The relative similarities in the results for the persistent, increased and decreased insecurity groups suggest that experiencing any recent or current instance of insecurity may impact on metabolic and inflammatory biomarkers, with potentially cumulative detrimental impacts. Evidence for potential cumulative effects of financial strain were demonstrated for the measures of inflammation and glucose tolerance, while for the measure of missed bill payments, a cumulative effect was suggested for GGT. Results were more mixed for the other biomarker outcomes, suggesting that perhaps the other metabolic markers do not operate in a cumulative manner. This is a first step towards identifying how repeated exposure to economic insecurity may become biologically embedded.

### Methodological considerations

The main limitation of our study, which impedes our ability to establish causality, is the lack of biomarker data at both time points. However, our analysis improves on the existing largely cross-sectional literature by examining change over time using two measures of economic insecurity and studying several biomarker outcomes. We also only accounted for potential confounding at baseline, and only had data on BMI and smoking for the whole sample at the time of biomarker measurement, so we cannot rule out the possibility of confounding due to differences in BMI or smoking at baseline, or the chance that changes to sociodemographic factors, such as employment status, were driving our findings. However, measures of kidney function, included as falsification tests, were relatively unaffected by economic insecurity and therefore provide some reassurance that findings are not due to residual confounding,^41^ and strengthens our confidence in the specificity of our findings. Although our analyses were weighted, selection bias may still be an issue. However, our results are likely to be more generalisable than previous studies, such as those using occupational cohorts.^29^ Further research that uses administrative income or wealth data is ideally required to compare objective and subjective measures. Furthermore, as a first step, we separately considered each biomarker, but it may be possible that the biomarkers included are casually related to one another.

### Implications

Experiencing economic insecurity during working-age may have important implications for health and well-being. Those with less economic resources are exposed to more periods of financial insecurity throughout their life course, with potentially cumulative impacts on health. Aside from its other benefits, the prevention of economic insecurity may have important public health effects. This is increasingly important as recent changes to welfare policy and increases in job insecurity are likely to cause increased financial strain among disadvantaged groups. Preventing economic insecurity via adequate social and employment protection policies is likely to have health benefits by reducing psychosocial stress, which could have important impacts on metabolic and inflammatory systems.

**What is already known on this subject**

- Economic insecurity is associated with adverse health outcomes, such as depression and coronary heart disease.
- To our knowledge, studies have yet to explore the association between longitudinal changes in economic insecurity and metabolic, inflammatory and liver function biomarkers, partly reflecting the lack of available data.

**What this study adds**

- We take advantage of the first nationally representative study of the working-age population of Great Britain to include blood analyte data and longitudinal measures of economic insecurity during a period of economic recession.
- Experiencing consistent economic insecurity is associated with worsened metabolic and inflammatory biomarkers (eg, high-density lipoprotein-cholesterol, triglycerides, glycated haemoglobin, C reactive protein (CRP) and fibrinogen).
- CRP and fibrinogen levels were 15.2% and 2.6% higher among those who experienced consistent financial strain, respectively, with evidence for a potential cumulative effect.
