# Supplementary material for: Economic insecurity during the Great Recession and metabolic, inflammatory and liver function biomarkers: analysis of the UK Household Longitudinal Study
Source: J Epidemiol Community Health. 2017 Aug 28;71(10):1005–13. doi: 10.1136/jech-2017-209105 (PMC5754862; doi:10.1136/jech-2017-209105)
Supplement: Supplementary data [file jech-2017-209105supp001.pdf]

Online supplementary material

Additional file 1: Further methodological details

Biomarkers

During the health assessment, non-fasting blood samples were taken from consenting eligible participants. It took 2.6 days on average for the blood samples to be processed in the laboratory, with 90% of samples being processed within four days (1). The blood samples were analysed by the Newcastle upon Tyne Hospitals NHS Foundations Trust (NUTH). Ethical approval from the National Research Ethics Service was obtained for the collection of biosocial data by trained nurses, further details can be found elsewhere (2).

Additional file 2

Table A1: Details of missing data for any variable with missing values

**Covariate**	N	%
Baseline employment status	19	0.3
Baseline education level	16	0.2
Baseline marital status	1	0.0
Baseline self-reported health	1	0.0
Baseline social class	346	1.0
Baseline mental health (GHQ)	752	10.1
BMI (time 2)	129	4.6
Current smoking (time 2)	24	0.3
Baseline economic insecurity (financial strain)	98	1.3
Baseline economic insecurity (missed bills)	11	0.1
Economic insecurity (change) (financial strain)	100	1.3
Economic insecurity (change) (missed bills)	19	0.3
**Outcome**		
Total cholesterol	195	2.6
HDL-cholesterol	205	2.7
Triglycerides	192	2.6
HbA1c	544	7.3
CRP	391	5.2
Fibrinogen	855	11.5
GGT	244	3.3
Creatinine	178	2.4
Urea	174	2.3

Table A2: Geometric means for each biomarker according to baseline economic insecurity

	Economic insecurity (financial strain)								Economic insecurity (missed bills)							
	Secure				Insecure				Secure				Insecure			
**Outcome**	**N**	**Mean**	**Lower 95% CI**	**Upper 95% CI**	**N**	**Mean**	**Lower 95% CI**	**Upper 95% CI**	**N**	**Mean**	**Lower 95% CI**	**Upper 95% CI**	**N**	**Mean**	**Lower 95% CI**	**Upper 95% CI**
**Total cholesterol** mmol/L	3,874	5.35	5.32	5.39	2,479	5.33	5.29	5.38	5,397	5.35	5.32	5.38	956	5.30	5.22	5.37
**HDL cholesterol** mmol/L	3,871	1.51	1.49	1.52	2,472	1.42	1.41	1.44	5,389	1.49	1.48	1.50	954	1.38	1.35	1.41
**Triglycerides** mmol/L	3,877	1.50	1.47	1.53	2,479	1.59	1.55	1.62	5,398	1.51	1.49	1.54	958	1.63	1.57	1.70
**HbA1c** mmol/mol	3,703	34.93	34.77	35.10	2,345	36.16	35.87	36.45	5,146	35.28	35.12	35.43	902	36.23	35.78	36.69
**CRP** mg/L	3,613	1.17	1.13	1.20	2,273	1.41	1.36	1.47	5,004	1.22	1.19	1.25	882	1.49	1.39	1.59
**Fibrinogen** g/L	3,553	2.58	2.56	2.59	2,231	2.68	2.66	2.71	4,921	2.60	2.59	2.62	863	2.70	2.67	2.74
**GGT** u/L	3,846	24.35	23.82	24.90	2,467	25.98	25.22	26.76	5,362	24.49	24.02	24.96	951	27.68	26.37	29.05
**Creatinine** umol/L	3,881	72.94	72.46	73.43	2,485	71.15	70.54	71.77	5,403	72.46	72.04	72.87	963	70.92	69.96	71.89
**Urea** mmol/L	3,882	5.78	5.74	5.83	2,487	5.54	5.48	5.61	5,406	5.72	5.68	5.76	963	5.50	5.41	5.60

HDL=High density lipoprotein; HbA1c=glycated haemoglobin; CRP=C-Reactive Protein; GGT= Gamma glutamyl transferase; Weighted geometric means

Table A3: Geometric means for each biomarker according to change in economic insecurity (using financial strain measure)

	**Total**				**Remained secure**				**Increased insecurity**				**Decreased insecurity**				**Remined insecure**			
**Outcome**	**N**	**Mean**	**Lower 95% CI**	**Upper 95% CI**	**N**	**Mean**	**Lower 95% CI**	**Upper 95% CI**	**N**	**Mean**	**Lower 95% CI**	**Upper 95% CI**	**N**	**Mean**	**Lower 95% CI**	**Upper 95% CI**	**N**	**Mean**	**Lower 95% CI**	**Upper 95% CI**
**Total cholesterol** mmol/L	6,353	5.34	5.32	5.37	3,179	5.36	5.32	5.40	695	5.31	5.22	5.39	625	5.33	5.24	5.42	1,854	5.34	5.28	5.39
**HDL cholesterol** mmol/L	6,343	1.47	1.46	1.48	3,177	1.53	1.51	1.54	694	1.43	1.40	1.46	624	1.47	1.44	1.51	1,848	1.41	1.39	1.43
**Triglycerides** mmol/L	6,356	1.53	1.51	1.56	3,181	1.47	1.44	1.50	696	1.60	1.53	1.67	625	1.54	1.47	1.61	1,854	1.60	1.56	1.65
**HbA1c** mmol/mol	6,048	35.44	35.29	35.60	3,031	34.93	34.75	35.10	672	34.95	34.53	35.39	592	35.33	34.82	35.84	1,753	36.43	36.09	36.78
**CRP** mg/L	5,886	1.26	1.23	1.30	2,973	1.14	1.10	1.18	640	1.31	1.21	1.41	573	1.22	1.12	1.32	1,700	1.48	1.41	1.55
**Fibrinogen** g/L	5,784	2.62	2.61	2.63	2,922	2.57	2.55	2.59	631	2.60	2.56	2.64	561	2.62	2.57	2.66	1,670	2.70	2.68	2.73
**GGT** u/L	6,313	25.03	24.59	25.48	3,156	24.15	23.58	24.75	690	25.20	23.87	26.60	622	24.47	23.09	25.94	1,845	26.47	25.58	27.40
**Creatinine** umol/L	6,366	72.18	71.80	72.56	3,185	72.93	72.39	73.46	696	73.02	71.91	74.15	626	71.38	70.14	72.64	1,859	71.08	70.38	71.78
**Urea** mmol/L	6,369	5.68	5.65	5.72	3,186	5.79	5.75	5.84	696	5.74	5.63	5.84	626	5.65	5.54	5.77	1,861	5.51	5.44	5.58

HDL=High density lipoprotein; HbA1c=glycated haemoglobin; CRP=C-Reactive Protein; GGT= Gamma glutamyl transferase; Weighted geometric means

Table A4: Geometric means for each biomarker according to economic insecurity (using missed bills measure)

	Total				Remained secure				Increased insecurity				Decreased insecurity				Remained insecure			
**Outcome**	**N**	**Mean**	**Lower 95% CI**	**Upper 95% CI**	**N**	**Mean**	**Lower 95% CI**	**Upper 95% CI**	**N**	**Mean**	**Lower 95% CI**	**Upper 95% CI**	**N**	**Mean**	**Lower 95% CI**	**Upper 95% CI**	**N**	**Mean**	**Lower 95% CI**	**Upper 95% CI**
**Total cholesterol** mmol/L	6,353	5.34	5.32	5.37	4,965	5.35	5.32	5.38	432	5.41	5.31	5.52	493	5.35	5.25	5.46	463	5.24	5.14	5.35
**HDL cholesterol** mmol/L	6,343	1.47	1.46	1.48	4,958	1.49	1.48	1.51	431	1.47	1.42	1.52	491	1.40	1.37	1.44	463	1.36	1.32	1.40
**Triglycerides** mmol/L	6,356	1.53	1.51	1.56	4,967	1.51	1.49	1.54	431	1.54	1.45	1.63	495	1.63	1.55	1.72	463	1.63	1.54	1.73
**HbA1c** mmol/mol	6,048	35.44	35.29	35.60	4,738	35.26	35.10	35.43	408	35.40	34.86	35.96	471	35.74	35.17	36.32	431	36.72	36.01	37.44
**CRP** mg/L	5,886	1.26	1.23	1.30	4,609	1.20	1.17	1.24	395	1.41	1.28	1.55	461	1.37	1.25	1.49	421	1.61	1.46	1.77
**Fibrinogen** g/L	5,784	2.62	2.61	2.63	4,538	2.60	2.58	2.61	383	2.65	2.60	2.71	449	2.69	2.64	2.74	414	2.72	2.66	2.77
**GGT** u/L	6,313	25.03	24.59	25.48	4,934	24.31	23.84	24.80	428	26.45	24.72	28.31	489	26.36	24.62	28.22	462	28.98	27.06	31.04
**Creatinine** umol/L	6,366	72.18	71.80	72.56	4,970	72.44	72.01	72.87	433	72.67	71.23	74.15	497	71.71	70.36	73.08	466	70.18	68.82	71.57
**Urea** mmol/L	6,369	5.68	5.65	5.72	4,972	5.73	5.69	5.77	434	5.60	5.46	5.74	497	5.46	5.33	5.59	466	5.54	5.40	5.69

HDL=High density lipoprotein; HbA1c=glycated haemoglobin; CRP=C-Reactive Protein; GGT= Gamma glutamyl transferase; Weighted geometric means

Table A5: Associations between changes in economic insecurity (using missed bills measure) and metabolic, inflammatory, liver and kidney function biomarkers in Understanding Society

	**Total cholesterol**	**HDL-cholesterol**	**Triglycerides**	**HbA1c**	**CRP**	**Fibrinogen**	**GGT**	**Creatinine**	**Urea**
	Coeff.	Coeff.	Coeff.	Coeff.	Coeff.	Coeff.	Coeff.	Coeff.	Coeff.
	[95% CI]	[95% CI]	[95% CI]	[95% CI]	[95% CI]	[95% CI]	[95% CI]	[95% CI]	[95% CI]
**Model 1**									
increased insecurity [a]	1.024*	0.987	1.036	1.016	1.206***	1.032*	1.114**	1.003	0.984
	[1.000,1.048]	[0.952,1.024]	[0.971,1.105]	[0.999,1.032]	[1.084,1.342]	[1.007,1.058]	[1.035,1.199]	[0.984,1.023]	[0.957,1.013]
decreased insecurity	1.010	0.936***	1.101**	1.022*	1.156**	1.043***	1.114*	0.994	0.961**
	[0.987,1.033]	[0.909,0.965]	[1.035,1.171]	[1.005,1.038]	[1.047,1.275]	[1.020,1.065]	[1.020,1.218]	[0.977,1.012]	[0.934,0.988]
remained insecure	1.002	0.899***	1.143***	1.062***	1.381***	1.058***	1.296***	0.991	0.992
	[0.980,1.024]	[0.870,0.929]	[1.067,1.224]	[1.038,1.086]	[1.234,1.546]	[1.035,1.081]	[1.202,1.398]	[0.970,1.011]	[0.962,1.023]
**Model 2**									
increased insecurity [a]	1.026*	0.998	1.019	1.007	1.142*	1.022	1.083*	1.009	0.995
	[1.002,1.050]	[0.963,1.035]	[0.955,1.087]	[0.991,1.024]	[1.024,1.273]	[0.996,1.048]	[1.006,1.167]	[0.990,1.029]	[0.968,1.024]
decreased insecurity	1.011	0.948***	1.079*	1.015	1.095	1.032**	1.078	1.000	0.969*
	[0.989,1.034]	[0.921,0.977]	[1.016,1.147]	[0.998,1.032]	[0.993,1.208]	[1.010,1.054]	[0.989,1.174]	[0.982,1.018]	[0.942,0.997]
remained insecure	1.003	0.914***	1.106**	1.049***	1.260***	1.039***	1.238***	0.999	1.004
	[0.980,1.026]	[0.884,0.945]	[1.032,1.184]	[1.025,1.074]	[1.122,1.414]	[1.016,1.062]	[1.145,1.339]	[0.979,1.020]	[0.974,1.036]
**Model 3**									
increased insecurity [a]	1.025*	0.999	1.020	1.008	1.139*	1.020	1.082*	1.011	0.997
	[1.002,1.050]	[0.964,1.035]	[0.957,1.087]	[0.992,1.024]	[1.022,1.270]	[0.995,1.046]	[1.005,1.166]	[0.991,1.031]	[0.970,1.026]
decreased insecurity	1.011	0.952**	1.078*	1.014	1.085	1.029**	1.074	1.002	0.972*
	[0.989,1.034]	[0.924,0.981]	[1.015,1.144]	[0.998,1.031]	[0.984,1.197]	[1.007,1.051]	[0.986,1.170]	[0.984,1.020]	[0.946,0.999]
remained insecure	1.003	0.917***	1.111**	1.049***	1.250***	1.034**	1.237***	1.005	1.012
	[0.980,1.025]	[0.887,0.949]	[1.036,1.190]	[1.025,1.074]	[1.115,1.401]	[1.011,1.057]	[1.144,1.337]	[0.984,1.027]	[0.981,1.044]
**Model 4**									
increased insecurity [a]	1.026*	1.001	1.016	1.005	1.129*	1.019	1.075	1.011	0.998
	[1.002,1.050]	[0.966,1.038]	[0.953,1.083]	[0.990,1.021]	[1.011,1.260]	[0.994,1.044]	[0.998,1.158]	[0.992,1.031]	[0.970,1.026]
decreased insecurity	1.012	0.956**	1.070*	1.010	1.071	1.027*	1.062	1.002	0.973
	[0.990,1.035]	[0.927,0.986]	[1.007,1.137]	[0.994,1.027]	[0.970,1.182]	[1.005,1.049]	[0.976,1.156]	[0.985,1.020]	[0.946,1.000]
remained insecure	1.005	0.926***	1.091*	1.040***	1.207**	1.028*	1.203***	1.006	1.014
	[0.982,1.028]	[0.896,0.958]	[1.019,1.169]	[1.016,1.064]	[1.077,1.352]	[1.006,1.051]	[1.113,1.300]	[0.985,1.027]	[0.983,1.046]
**Model 5**									

increased insecurity [a]	1.025*	1.002	1.013	1.005	1.130*	1.019	1.074	1.011	0.999
	[1.002,1.050]	[0.967,1.038]	[0.950,1.080]	[0.989,1.021]	[1.012,1.261]	[0.994,1.045]	[0.996,1.157]	[0.991,1.031]	[0.971,1.027]
decreased insecurity	1.011	0.956**	1.067*	1.010	1.072	1.027*	1.060	1.002	0.974
	[0.989,1.035]	[0.927,0.986]	[1.003,1.134]	[0.994,1.027]	[0.971,1.184]	[1.005,1.049]	[0.973,1.155]	[0.984,1.020]	[0.947,1.001]
remained insecure	1.004	0.927***	1.086*	1.040***	1.209**	1.028*	1.200***	1.005	1.015
	[0.981,1.027]	[0.897,0.959]	[1.014,1.163]	[1.016,1.064]	[1.079,1.355]	[1.006,1.051]	[1.110,1.297]	[0.985,1.027]	[0.984,1.048]
N	6425	6417	6427	6162	6003	5905	6390	6437	6439

Linear regression models calculated using logged dependent variables, with exponentiated coefficients shown
95% confidence intervals in brackets; [a] reference is economically secure at both time points; HbA1c=glycated haemoglobin; CRP=C-Reactive Protein; GGT= Gamma glutamyl transferase; * $p < 0.05$, ** $p < 0.01$, *** $p < 0.001$

Model 1: Adjusted for age and sex; Model 2: Model 1 + education level, marital status, employment status, social class; Model 3=Model 2 + household income quintile; Model 4=Model 3 + limiting long-standing illness, self-reported health; Model 5=Model 4 + GHQ caseness

Table A6: Associations between changes in economic insecurity and metabolic, inflammatory, liver and kidney function biomarkers in Understanding Society, mutually adjusted for both measures of economic insecurity

	**Total cholesterol**	**HDL-cholesterol**	**Triglycerides**	**HbA1c**	**CRP**	**Fibrinogen**	**GGT**	**Creatinine**	**Urea**
	Coeff.	Coeff.	Coeff.	Coeff.	Coeff.	Coeff.	Coeff.	Coeff.	Coeff.
**Financial strain**	[95% CI]	[95% CI]	[95% CI]	[95% CI]	[95% CI]	[95% CI]	[95% CI]	[95% CI]	[95% CI]
increased insecurity [a]	1.000	0.957**	1.074*	1.000	1.104*	1.006	1.009	1.006	1.005
	[0.981,1.020]	[0.930,0.984]	[1.015,1.135]	[0.986,1.013]	[1.003,1.216]	[0.986,1.028]	[0.946,1.075]	[0.991,1.022]	[0.983,1.027]
decreased insecurity	1.005	0.981	1.038	1.008	1.009	1.008	0.990	0.997	1.001
	[0.985,1.025]	[0.953,1.009]	[0.981,1.099]	[0.991,1.024]	[0.914,1.114]	[0.987,1.030]	[0.928,1.055]	[0.980,1.014]	[0.978,1.026]
remained insecure	1.004	0.956***	1.036	1.021**	1.118**	1.021*	0.988	0.999	0.983
	[0.988,1.020]	[0.935,0.978]	[0.993,1.082]	[1.008,1.034]	[1.035,1.208]	[1.005,1.037]	[0.939,1.040]	[0.985,1.012]	[0.964,1.002]
**Missed bills**									
increased insecurity [a]	1.025*	1.009	1.007	1.002	1.108	1.016	1.075	1.011	1.001
	[1.001,1.050]	[0.974,1.046]	[0.944,1.075]	[0.986,1.019]	[0.992,1.237]	[0.991,1.042]	[0.997,1.158]	[0.991,1.031]	[0.973,1.030]
decreased insecurity	1.011	0.963*	1.060	1.007	1.055	1.023*	1.063	1.003	0.977
	[0.988,1.034]	[0.933,0.994]	[0.996,1.129]	[0.990,1.024]	[0.954,1.166]	[1.001,1.045]	[0.975,1.158]	[0.985,1.021]	[0.950,1.004]
remained insecure	1.003	0.939***	1.077*	1.033**	1.168**	1.021	1.205***	1.006	1.022
	[0.980,1.026]	[0.907,0.973]	[1.004,1.155]	[1.009,1.057]	[1.039,1.313]	[0.999,1.045]	[1.112,1.305]	[0.984,1.028]	[0.990,1.055]

Linear regression models calculated using logged dependent variables, with exponentiated coefficients shown
95% confidence intervals in brackets; [a] reference is economically secure at both time points; HbA1c=glycated haemoglobin; CRP=C-Reactive Protein; GGT= Gamma glutamyl transferase; * $p < 0.05$, ** $p < 0.01$, *** $p < 0.001$

Models adjusted for age, sex, education level, marital status, employment status, social class, household income quintile, limiting long-standing illness, self-reported health, GHQ caseness

Table A7: The association between the accummulation of economic insecurity (using missed bills measure) and metabolic, inflammatory, liver and kidney function biomarkers in Understanding Society

	**Total cholesterol**	**HDL-cholesterol**	**Triglycerides**	**HbA1c**	**CRP**	**Fibrinogen**	**GGT**	**Creatinine**	**Urea**
	Coeff.	Coeff.	Coeff.	Coeff.	Coeff.	Coeff.	Coeff.	Coeff.	Coeff.
	[95% CI]	[95% CI]	[95% CI]	[95% CI]	[95% CI]	[95% CI]	[95% CI]	[95% CI]	[95% CI]
1[a]	1.016	0.976	1.039	1.005	1.106*	1.029**	1.063	1.008	0.977*
	[0.998,1.035]	[0.950,1.004]	[0.986,1.096]	[0.992,1.018]	[1.016,1.205]	[1.010,1.048]	[0.993,1.137]	[0.993,1.024]	[0.955,0.999]
2	1.005	0.994	1.040	1.024	1.063	0.999	1.092	0.994	0.998
	[0.975,1.037]	[0.954,1.035]	[0.961,1.127]	[0.999,1.050]	[0.913,1.237]	[0.970,1.028]	[0.991,1.204]	[0.970,1.018]	[0.959,1.039]
3	1.000	0.895***	1.049	1.035*	1.226*	1.055**	1.159**	0.998	1.015
	[0.965,1.036]	[0.850,0.943]	[0.940,1.172]	[1.007,1.063]	[1.031,1.457]	[1.019,1.092]	[1.038,1.293]	[0.971,1.027]	[0.966,1.065]
4	1.006	0.907***	1.121**	1.037	1.216*	1.011	1.261***	1.024	1.023
	[0.975,1.038]	[0.864,0.952]	[1.029,1.223]	[0.998,1.078]	[1.034,1.429]	[0.978,1.045]	[1.118,1.422]	[0.990,1.058]	[0.977,1.071]
N	6385	6377	6387	6124	5963	5866	6350	6396	6398

Linear regression models calculated using logged dependent variables, with exponentiated coefficients shown

95% confidence intervals in brackets; [a] reference is no economic insecurity; HbA1c=glycated haemoglobin; CRP=C-Reactive Protein; GGT= Gamma glutamyl transferase; * $p < 0.05$, ** $p < 0.01$, *** $p < 0.001$

Models adjusted for age, sex, education level, marital status, employment status, social class, household income quintile, limiting long-standing illness, self-reported health, GHQ caseness

Table A8: Results from linear regression models investigating the influence of economic insecurity (using financial strain measure) and metabolic, inflammatory, liver and kidney function biomarkers with additional inclusion of BMI and smoking

	**Total cholesterol**	**HDL-cholesterol**	**Triglycerides**	**HbA1c**	**CRP**	**Fibrinogen**	**GGT**	**Creatinine**	**Urea**
	Coeff.	Coeff.	Coeff.	Coeff.	Coeff.	Coeff.	Coeff.	Coeff.	Coeff.
	[95% CI]	[95% CI]	[95% CI]	[95% CI]	[95% CI]	[95% CI]	[95% CI]	[95% CI]	[95% CI]
**Model 1**									
increased insecurity [a]	0.999	0.961**	1.064*	1.000	1.093	1.006	1.007	1.006	1.004
	[0.981,1.019]	[0.937,0.986]	[1.008,1.122]	[0.987,1.014]	[0.999,1.196]	[0.985,1.027]	[0.944,1.074]	[0.990,1.021]	[0.982,1.026]
decreased insecurity	1.005	0.980	1.038	1.007	1.001	1.007	0.992	0.996	1.000
	[0.985,1.025]	[0.955,1.005]	[0.985,1.094]	[0.991,1.024]	[0.915,1.095]	[0.987,1.028]	[0.932,1.056]	[0.979,1.014]	[0.976,1.025]
remained insecure	1.002	0.962***	1.020	1.023***	1.086*	1.019*	0.995	0.997	0.982
	[0.987,1.018]	[0.943,0.981]	[0.980,1.061]	[1.010,1.035]	[1.012,1.166]	[1.003,1.034]	[0.948,1.045]	[0.985,1.010]	[0.964,1.001]
**Model 2**									
increased insecurity [a]	0.998	0.964**	1.059*	0.999	1.086	1.002	1.000	1.007	1.007
	[0.979,1.018]	[0.940,0.989]	[1.004,1.117]	[0.985,1.013]	[0.992,1.188]	[0.982,1.023]	[0.937,1.066]	[0.991,1.022]	[0.985,1.029]
decreased insecurity	1.004	0.983	1.034	1.006	0.995	1.005	0.986	0.997	1.003
	[0.984,1.024]	[0.958,1.008]	[0.981,1.090]	[0.990,1.023]	[0.910,1.089]	[0.985,1.025]	[0.927,1.050]	[0.980,1.015]	[0.978,1.027]
remained insecure	1.001	0.966***	1.014	1.021***	1.077*	1.014	0.986	0.998	0.986
	[0.985,1.016]	[0.947,0.986]	[0.975,1.055]	[1.009,1.033]	[1.004,1.156]	[0.999,1.030]	[0.939,1.035]	[0.986,1.011]	[0.967,1.005]
N	6425	6417	6427	6162	6003	5905	6390	6437	6439

Exponentiated coefficients; 95% confidence intervals in brackets; [a] reference is economically secure at both time points; HbA1c=glycated haemoglobin; CRP=C-Reactive Protein; GGT= Gamma glutamyl transferase; * $p < 0.05$, ** $p < 0.01$, *** $p < 0.001$

Model 1: adjusted for age, sex, education level, marital status, employment status, social class, household income quintile, limiting long-standing illness, self-reported health, GHQ caseness and BMI; Model 2: Model 1 and current smoking

Table A9: Results from linear regression models investigating the influence of economic insecurity (using missed bills measure) and metabolic, inflammatory, liver and kidney function biomarkers with additional inclusion of BMI and smoking

	**Total cholesterol**	**HDL-cholesterol**	**Triglycerides**	**HbA1c**	**CRP**	**Fibrinogen**	**GGT**	**Creatinine**	**Urea**
	Coeff.	Coeff.	Coeff.	Coeff.	Coeff.	Coeff.	Coeff.	Coeff.	Coeff.
	[95% CI]	[95% CI]	[95% CI]	[95% CI]	[95% CI]	[95% CI]	[95% CI]	[95% CI]	[95% CI]
**Model 1**									
increased insecurity [a]	1.025*	1.000	1.015	1.006	1.126*	1.019	1.078*	1.011	0.998
	[1.002,1.049]	[0.968,1.034]	[0.954,1.080]	[0.991,1.022]	[1.013,1.251]	[0.995,1.044]	[1.002,1.159]	[0.991,1.030]	[0.971,1.027]
decreased insecurity	1.009	0.969*	1.042	1.007	1.016	1.020	1.038	1.000	0.972*
	[0.987,1.032]	[0.941,0.997]	[0.983,1.105]	[0.990,1.023]	[0.925,1.116]	[0.999,1.041]	[0.955,1.129]	[0.983,1.019]	[0.946,1.000]
remained insecure	1.000	0.943***	1.054	1.037**	1.138*	1.020	1.170***	1.002	1.013
	[0.978,1.023]	[0.913,0.973]	[0.988,1.125]	[1.014,1.060]	[1.024,1.264]	[0.999,1.042]	[1.086,1.260]	[0.982,1.023]	[0.982,1.045]
**Model 2**									
increased insecurity [a]	1.023*	1.005	1.009	1.005	1.118*	1.015	1.068	1.012	1.002
	[1.000,1.047]	[0.971,1.039]	[0.949,1.073]	[0.990,1.021]	[1.005,1.243]	[0.991,1.040]	[0.995,1.147]	[0.992,1.032]	[0.974,1.031]
decreased insecurity	1.009	0.970*	1.041	1.006	1.014	1.019	1.036	1.001	0.973
	[0.986,1.032]	[0.943,0.997]	[0.982,1.104]	[0.990,1.023]	[0.924,1.113]	[0.999,1.040]	[0.952,1.128]	[0.983,1.019]	[0.947,1.001]
remained insecure	0.997	0.950**	1.044	1.034**	1.121*	1.013	1.153***	1.005	1.020
	[0.975,1.020]	[0.920,0.980]	[0.978,1.114]	[1.011,1.058]	[1.009,1.245]	[0.991,1.034]	[1.070,1.242]	[0.984,1.026]	[0.989,1.052]
N	6425	6417	6427	6162	6003	5905	6390	6437	6439

Exponentiated coefficients; 95% confidence intervals in brackets; [a] reference is economically secure at both time points; HbA1c=glycated haemoglobin; CRP=C-Reactive Protein; GGT= Gamma glutamyl transferase; * $p < 0.05$, ** $p < 0.01$, *** $p < 0.001$

Model 1: adjusted for age, sex, education level, marital status, employment status, social class, household income quintile, limiting long-standing illness, self-reported health, GHQ caseness and BMI; Model 2: Model 1 and current smoking

Figure A1: Economic security and metabolic, inflammatory, liver and kidney function biomarkers excluding those taking specific medications, coefficients are exponentiated

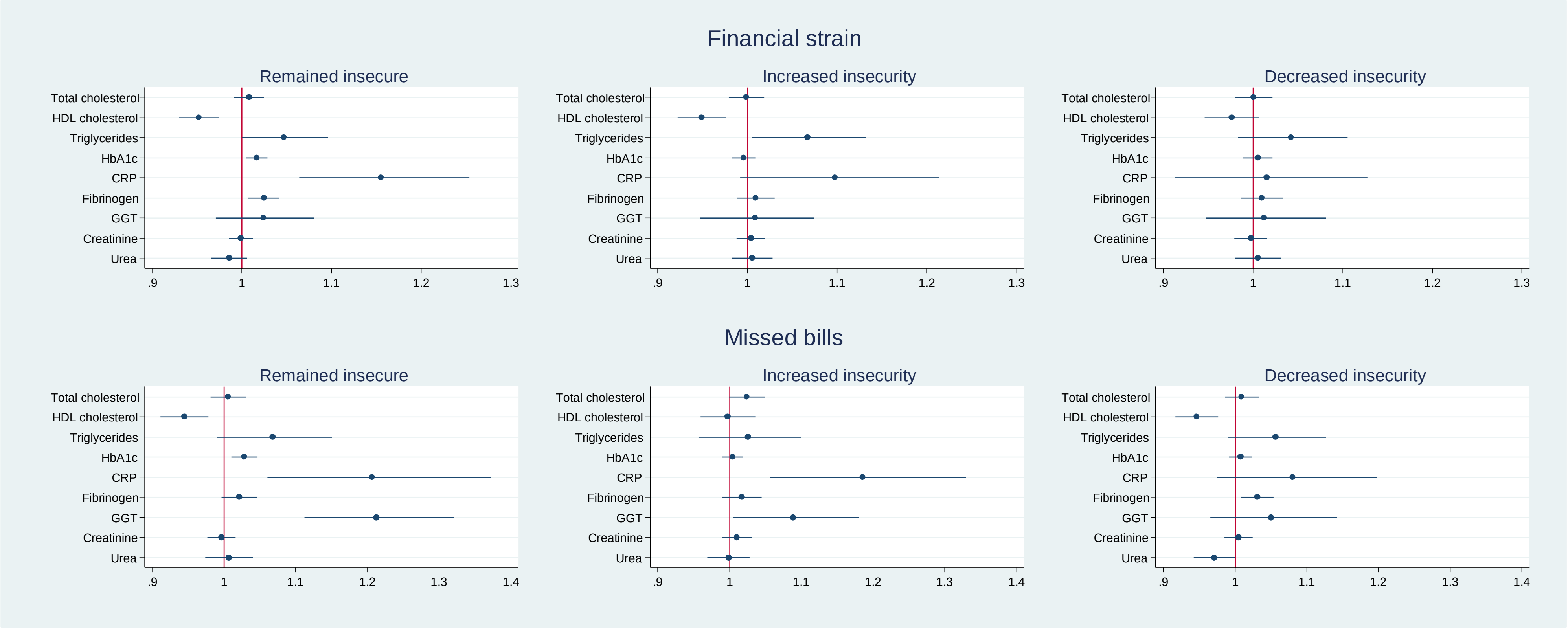

Figure A2: Economic security and metabolic, inflammatory, liver and kidney function, coefficients are exponentiated (sample restricted to individuals with complete biomarker data for each outcome)

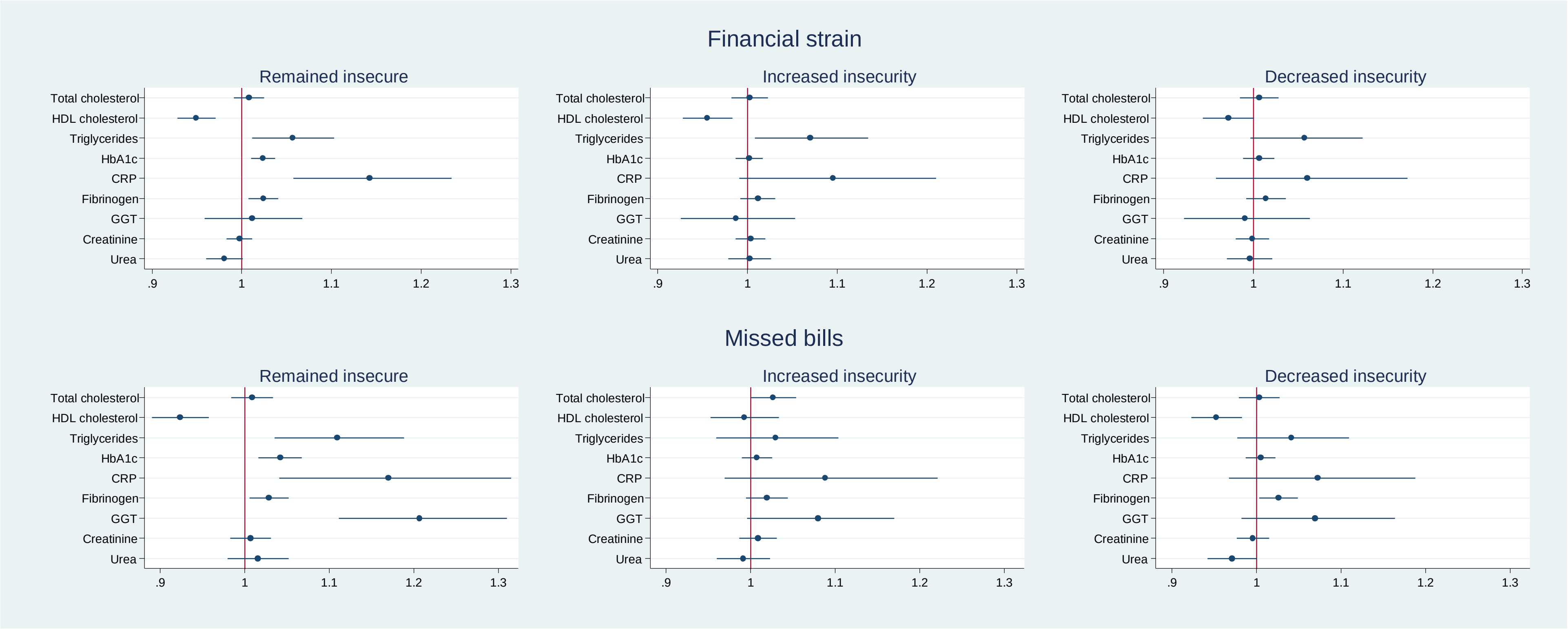

References

1. Benzeval M, Davillas A, Kumari M, Lynn P. Understanding Society: The UK Household Longitudinal Study Biomarker User Guide and Glossary: Institute for Social and Economic Research, University of Essex, Colchester2014.

2. McFall S, Petersen J, Kaminska O, Lynn P. Understanding Society—The UK Household Longitudinal Study: Waves 2 and 3 Nurse Health Assessment, 2010–2012 Guide to Nurse Health Assessment. Institute for Social and Economic Research, University of Essex. 2013.
